# Safety Behaviors to Reduce Risk of Using Chemical Household Products: An Application of the Risk Perception Attitude Framework

**DOI:** 10.3390/ijerph17051528

**Published:** 2020-02-27

**Authors:** Minjung Lee, Myoungsoon You

**Affiliations:** 1 Department of Public Health Science, Graduate School of Public Health, Seoul National University, Seoul 08826, Korea; minjunglee23@gmail.com; 2 Institute of Health and Environment, Seoul National University, Seoul 08826, Korea

**Keywords:** chemical household products, risk perception, efficacy belief, safety behaviors, socioeconomic status, risk communication

## Abstract

Various chemical household products (CHPs) can make life more convenient; however, CHP users have higher rates of chemical exposure and are faced with the risk of accidents associated with using these products. Safe use of CHPs requires that individuals perform safety-related behaviors such as reading and following CHP risk information. As such, it may be worthwhile to apply the Risk Recognition Attitude (RPA) framework to classify groups of CHP users and investigate whether there is a difference in the safety behaviors between them. Therefore, the objectives of this study are to (a) examine social determinants of each group in the RPA framework, (b) identify different policies that would be effective for each group, and (c) provide evidence to inform the development of effective policies and risk communication strategies that encourage safety behaviors. The study included 1537 subjects and used an ANOVA with a post-hoc Tukey test to examine practices of the four groups in terms of two safety behaviors. A multinomial logistic regression was performed to identify factors that influence the classification of the group types. The results confirmed that safety behaviors associated with using CHPs differed according to weak levels of efficacy beliefs. Two groups of particular concern for low rates of safety behaviors were those with lower education and income levels. Recommendations include (a) customized safety policies and risk communications based on RPA characteristics, (b) distinctive messaging for different groups, (c) policy support for vulnerable populations, and (d) implementing ‘user-centered’ rather than ‘substance-centered’ policies and communications for the public.

## 1. Introduction

Consuming chemicals is inevitable, as these agents appear in many consumer products, including cosmetics, cleaning agents, pesticides, solvents, and coatings. Such chemicals can make life more convenient, but individuals who use them may have a higher rate of exposure to chemicals [1,2]. Chemicals in chemical household products (CHPs) include phthalates (e.g., plasticizers), oxybenzone (e.g., sunscreen), triclosan (e.g., antibacterial/antifungal), heavy metals (e.g., lead and arsenic), nitrosamines (an ingredient in cured meats), hydroquinone (e.g., depigmenting agent), and 1,4-dioxane. Such chemicals can be absorbed via inhalation, ingestion, or dermal absorption, and this exposure can induce adverse health effects, including skin rashes, allergies, eye irritation, endocrine disruption, neurotoxicity, birth defects, or cancers [3,4,5,6]. Numerous studies have shown that substances mimicking estrogen (e.g., bisphenol A) in the blood of almost all CHP users are not only detectable but also maintain a constant level [7,8].

The risk of using CHPs is not limited to exposure to chemicals; there is also a risk of accidents during use. In Korea, as the consumption of CHPs increases, the number of accidents related to CHPs is also increasing [9,10]. Some accidents due to CHPs are fatal, as evidenced by 174 deaths in Korea attributed to the use of humidifier disinfectants containing PHMG (polyhexamethylene guanidine) and PGH (Oligo(2-(2-ethoxy) ethoxyethyl guanidinium chloride)), which the Korea Centers for Disease Control & Prevention (KCDC) confirmed had inhalation toxicity [11]. This tragedy outraged the public, leading to growing anxiety and concern for CHP safety. 

Nevertheless, the use of CHPs in everyday life is inevitable. Therefore, efforts of governments, CHP manufacturers, and environmental health researchers to ensure safe production, distribution, and use of CHPs are critical. In Korea, the Act on the Registration and Evaluation of Chemicals in Korea (K-REACH) passed on 30 April 2013 and was enacted on 1 January 2015 to protect the environment and public health via registering and screening of hazardous chemicals. This regulation aimed at reducing human health risks associated with CHP use by applying safety limits or labeling standards for dangerous chemical substances in these products. Even with these public controls to improve safety, individuals must also take measures themselves to reduce their risk of adverse health effects from chemical exposure and accidents related to CHP use. CHP users must make risk-management decisions concerning the safe use of CHPs, including how to choose, store, and use these products [12]. Risk information, such as warnings or guidelines, appear in the form of labels, instructions, and/or pictograms on CHP containers to raise awareness of hazards; they also provide guidance for implementing suitable risk management behaviors [2,13]. This information can help individuals make better “informed” risk-management decisions [14]. Certain regulations require product risk information to be written or printed clearly on the product package or container, and an understanding of risk information messages by users is a fundamental pillar of chemical legislation [15]. The U.S. Environmental Protection Agency (EPA) recommends that the public should read and follow product-warning labels that list risk information when using CHPs [16]. Similarly, since the “K-REACH” passed in 2013, chemical manufacturers and sellers must provide risk information on their products, and individuals are encouraged to read and follow it. There is a need for policies and efforts to compel individuals to read and follow product risk information while CHP use is taking on increasing importance. However, research evaluating the success of campaigns aimed at reducing accidents that occur in households is limited [17].

Risk information provides no benefit unless the user actually reads the information. Risk information conveyed by warnings often fails to reach users [18], and several previous studies have mentioned reasons for such communication failures. First, people do not read risk information on products that they perceive to be safe or familiar [19]. Several studies found that participants reported that they would be more likely to look for and read warnings on products judged to be more hazardous and less familiar [20,21]. Second, risk information might not be perceived as personally relevant. There is evidence that individuals are more likely to attend to other marketing communications when they are personally relevant [22,23]. Third, individuals simply may not read the information. Risk information presented after a set of procedural instructions may go unnoticed because people quit reading the risk information before seeing the warning [18,24]. Fourth, individuals may already be familiar with the risk information presented on the product or in its packaging. Individuals already familiar with the nature and content of risk information may not expend the effort required to heed a warning once its contents are known or perceived to be known.

After reading the risk information, not every individual follows information like safety instructions. Based on findings from previous studies, it is evident that people evaluate the same risks differently, since people assess risks subjectively based on their own experience [25], knowledge [26], and risk information gained from various sources [27]. Product characteristics [28], the contents of the information provided with the products [29], and recall of the risk information [30] also influence actual product use behaviors related to safety. However, to our knowledge, empirical studies based on theoretical frameworks that identify the factors that influence users’ compliance to risk information remains limited.

Public perception of the importance of reading and following risk information during CHP use, the effectiveness of national efforts, and the extent to which the public implements such practices remain unclear. Risk communication that encourages safe CHP use should consider the needs of the public, which often goes ignored in policy decisions [31]. Therefore, characterizing public perceptions of safety behaviors and the willingness of individuals to read and follow risk information before CHP use is critical. Moreover, identifying factors that affect safety behaviors is essential for establishing risk communication strategies and policy measures for the safe use of CHPs [32]. For example, public segmentation can aid governments and businesses in effectively responding to the public’s diverse needs and plays a significant role in managing user-related issues [32]. Providing different communication methods and customized messages for each category of individuals can maximize policy execution efficiency [33,34,35].

Two psychological constructs have the potential to determine the safety behavior of individuals using chemical products. The first, perceived risk, refers to an individual’s perception of vulnerability to a particular risk [36]. The second, efficacy, is the extent to which people believe that they can take action to avoid risk [37,38]. Perceived risk is a significant predictor of self-protective behavior [39,40,41,42,43,44,45,46]. Many health behavior models, such as the Protection Motivation Theory (PMT) [39] and Health Belief Model (HBM) [40], propose that risk perception and other concepts (e.g., perceived benefits and barriers) are key contributors to people’s willingness to make behavioral changes. Although researchers have hypothesized that a causal relationship exists between perceived risk and behavioral action [41,42,43,44,45,46], the evidence for this proposition is ambiguous since some studies have not identified a link between perceived risk and adoption of healthy behaviors [47,48,49,50]. Conversely, other studies have shown a negative correlation between perceived risk and practicing healthy behaviors [51,52]. Indeed, previous research confirms that not everyone has the same response to health risks, and risk perceptions alone cannot explain health behaviors.

**Figure 1:**
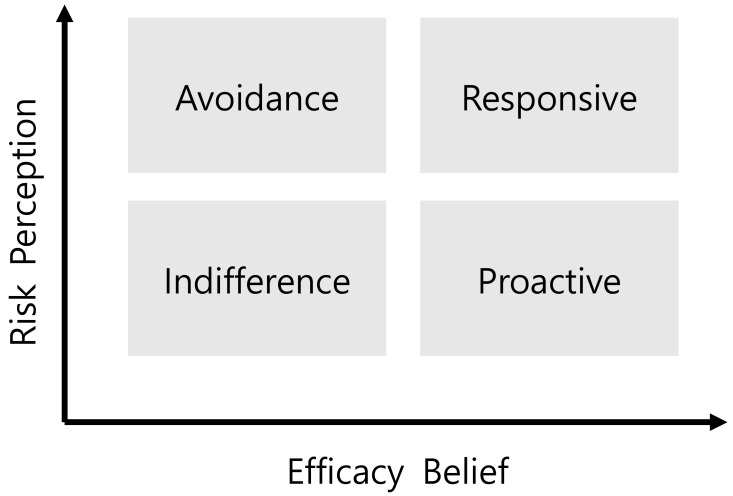
Risk Perception Attitude Framework [53].

The Risk Perception Attitude (RPA) framework [53], which hypothesizes that the efficacy beliefs of an individual act as a moderating variable between perceived risk and health behavior, offers one explanation for these conflicting results. The RPA framework was developed to explain how these constructs might be used to identify groups of people who could be targeted for health campaigns; the framework also focuses on the effects of an individual’s perceived risk and efficacy. The objective of the RPA framework is to target interventions and educational materials based on the type of individual as classified into one of four RPA groups (Figure 1). These four groups include the following: (1) “Responsive” individuals have high perceived risk and self-efficacy; (2) “avoidant” individuals have high perceived risk and low self-efficacy; (3) “proactive” individuals have low perceived risk and high self-efficacy; and (4) “indifferent” individuals have low perceived risk and low self-efficacy.

Individuals in the responsive group and avoidance group, with high levels of perceived risk, are highly motivated to avoid risk. However, these two attitude groups show different behavior patterns, given their varying degrees of efficacy beliefs. Individuals in the responsive group are individuals with a high sense of perceived risk and a high degree of efficacy belief for overcoming risks. Therefore, individuals in the responsive group are likely to exhibit health behaviors that are more desirable than any other group. On the other hand, individuals in the avoidance group have a risk perception equal to those in the responsive group, although their efficacy belief is weak, making them less motivated to act to reduce risk. In the RPA model, even in the same high level of perceived risk group, differences in behavior outcomes exist between the responsive and avoidance groups. In other words, RPA emphasizes that efficacy beliefs are essential for preventive behavior to protect against risk. Multiple studies have tested the utility of the RPA framework on health behaviors related to prevention of HIV, HPV [54], cancer [55], and diabetes [56,57], as well as preventive behaviors related to nutrition [55], maternal physical activity [58], and safety behavior while driving [59].

There are two primary objectives of this study. The first is to apply and test the RPA framework in the context of safety behaviors such as reading and following warning information for the use of CHPs and identify different policies and communication needs of each group. The second is to examine CHP use patterns and social determinants of group classification with the RPA framework for understanding group characteristics to enhance effective communication and foster development of customized messages and educational materials.

## 2. Methods

### 2.1. Data Collection

Data were collected through a survey conducted by Gallup Korea, a professional survey agent, during February 2018. Respondents included 1537 adult residents of Korea who were 20–65 years of age. We used proportionate quota sampling to characterize age, gender, and residence. Surveys were conducted in person. We excluded incomplete surveys from the analysis. The survey and consent to participate were approved by the Seoul National University Institutional Review Board (IRB No.1711/003-031).

### 2.2. The Questionnaire

#### 2.2.1. RPA Categories: Perceived Risk of Accident during CHP Use

Following Rimal and Juon (2010), perceived risk of accidents during the use of CHPs comprised both perceived susceptibility, which signifies individuals’ beliefs about their vulnerability to accidents, and perceived severity, which signifies the seriousness of an accident [60]. Respondents were asked, “What do you think is the risk of a possible accident during the use of CHPs?” and “What do you think is the risk of an accident’s severity during the use of CHPs?”. Responses were rated on a 5-point Likert-type scale (1 = not at all to 5 = extremely). The perceived risk was calculated as the average of perceived susceptibility and perceived severity.

#### 2.2.2. RPA Categories: Efficacy Belief of Accident Prevention during CHP Use

According to social cognitive theory [61], and following Rimal and Juon (2010), efficacy beliefs comprise (a) self-efficacy, or an individuals’ perceived ability to exert personal control, and (b) response efficacy or outcome expectations, which are the perceived benefits from engaging in a particular behavior. For self-efficacy, we used the questionnaire developed by Sherer [62] and five items that were highly reliable in the preliminary survey (e.g., ”Failure just makes me try harder”, “If I can’t do a job the first time, I keep trying until I can”) [63]. For response efficacy, respondents responded to the following statement: “Reading warning information before using CHPs is an effective way to reduce the risk of accident during the use of the product.” Responses for both constructs used a 5-point Likert-type scale (1 = not at all to 5 = extremely). Efficacy beliefs were calculated as the average of self-efficacy and response efficacy.

#### 2.2.3. Safety Behavior: Reading and Compliance with Risk Information during CHP Use

Participants responded to two items measuring the frequency with which they engage in two safety behaviors to reduce their risk during CHP use. One item measured whether the participants read risk information before using CHPs (e.g., “Before you use CHPs, do you read the risk information which is provided with the product?”). The other item assessed compliance with risk information accompanying the product (“When you use CHPs, do you follow the guidelines included in the risk information?). Responses used a 5-point Likert-type scale (1 = never, 3 = moderately often, and 5 = always).

#### 2.2.4. CHP Use Patterns

For CHP use patterns, we asked about the frequency of use for three CHP categories: Cleaning products (e.g., household bleach), pesticides, and laundry detergents. These three categories are classified as ‘risk-concerned products’ by the Korean government. We used a 7-point scale and assigned points as follows: “Rarely” (1 point), “2–3 times a month” (2 points), “1–2 times a week” (3 points), “3–4 times a week” (4 points), “5–6 times a week” (5 points), “every day” (6 points) and “more than two times a day” (7 points). Assuming that the CHP use patterns vary, we made an operational definition of “CHP use patterns” as the most frequent response among the three categories, instead of the average value, to account for variations in CHP usage patterns among a population [64,65].

#### 2.2.5. Sociodemographic Characteristics CHP Use Frequencies

Gender, age, education, monthly household income, job status, and presence of preschool children in the home were the independent variables (Table 1). For education, we assigned points as follows: “Middle school graduate” (1 point), “high school graduate” (2 points), “college graduate” (3 points), and “education beyond college (4 points)”. For monthly household income, we assigned between one and five points for incomes as follows: “Under 299 South Korean 10,000 won” (1 point) to “over 600 South Korean 10,000 won” (5 points). The presence of preschool children in the household was coded 1 = yes and 0 = none.

### 2.3. Formation of Four Risk Perception Attitude Groups

We used perceived risk and efficacy scores to classify respondents into four RPA groups for safety behaviors in terms of reading and compliance with risk information during CHP use. The four RPA groups included avoidant, proactive, indifferent, and responsive (see Figure 1). We used Sullivan et al. and Jo & Yoo’s [55,66] approach to segment the sample into RPA groups. A median split of both perceived risk and efficacy beliefs allowed for creating four groups. Here, we classified study participants based on median values for perceived risk and efficacy beliefs. As the median value of perceived risk was 4.0, the groups were classified as high (≥4.0) and low (<4.0) perceived risk groups. For efficacy belief, the median value was 3.6; therefore, we split the respondents as high (≥3.6) and low (<3.6) efficacy belief groups. After that, we formed a bi-plot with quadrants representing each risk classification. Participants in the upper right quadrant had both high perceived risk and high efficacy belief (“response” group) (see Figure 1), those in the upper left quadrant had high perceived risk and low efficacy belief (“avoidance” group), and likewise for other RPA classifications based on their location. Chi-square test was conducted to check whether the group classification was statistically significant.

### 2.4. Statistical Analysis

After classifying respondents based on their perceived risk and efficacy belief, we performed a multinomial logistic regression to reveal factors that influence the classification of RPA group types, and used an ANOVA with a post-hoc Tukey test to examine the average differences among four groups for two safety behaviors during CHP use. Statistical analyses were carried out using R version 3.5.1 (R Foundation for Statistical Computing, Vienna, Austria).

## 3. Results

### 3.1. Characteristics of the Study Population

Among the 1537 respondents, 787 (51.2%) were male, and 750 (48.8%) were female, with an average age of 42.5 years (Table 1). The majority of respondents (54.4%) had at least some college education, followed by those with only a high school education (41.2%). The most frequent monthly household income was 4.00–5.99 million won ($3500–$5200 USD; 45.3%), followed by 2.01–3.99 million won ($1700–$3450; 36.3%, Table 1). About 11.8% of the respondents reported having preschool children in their household.

### 3.2. Perceptions of Respondents

Perceived risk scores revealed that respondents perceived the risk of using CHPs as being higher than “moderately” (score = 3) yet lower than “very high” (score = 4) [*M* = 3.65, *SD* = 0.7]. The average score of the efficacy belief was also higher than “moderately” (score = 3) [*M* = 3.59, *SD* = 0.48]. The median was 4.0 for perceived risk and 3.6 for efficacy belief. Therefore, we divided the participants by the median values for high (≥4.0) and low (<4.0) perceived risk, high (≥3.6) and low (<3.6) efficacy belief, and then four groups of the RPA framework.

### 3.3. Safety Behavior and CHP Use Patterns

For CHP use patterns, most respondents reported using CHPs “1–2 times a week” (34.7%), and 7.6% of the respondents reported they use CHPs “more than 5–6 times a week.” CHP usage patterns varied; only 1.8% reported “always” (score = 5) reading CHP risk information before use, and 18.9% of the respondents reported they did so “often” (score = 4). For compliance behavior, only 1.0% reported “always” (score = 5) following the CHP risk information, and 33.2% reported doing so “often” (score = 4). We compared our results to those of a European study that investigated the same questions and found that a third of the respondents reported that they read the information (35%), whereas 30% stated they read the information most of the time [67]. As with compliance behavior, a third of the study sample in the European study reported following safety information while using CHPs “always” (36%) or doing so “most of the time” (35%) [67].

### 3.4. Classification of Individual Types According to the Level of Perceived Risk and Efficacy Beliefs

We categorized respondents into four RPA groups (i.e., avoidant, indifferent, proactive, and responsive) according to the level of perceived risk and efficacy beliefs (Table 2). We classified 434 (28.2%) participants as responsive (high perceived risk/high efficacy), 425 (27.7%) as indifferent (low perceived risk/low efficacy), 344 (22.4%) as avoidant (high perceived risk/low efficacy), and 334 (21.7%) as proactive (low perceived risk/high efficacy). Among the four groups, the number in the responsive group was relatively high, and the χ^2^ value for the group classification was 20.8 (*p <* 0.001), which was statistically significant.

### 3.5. Differences in Safety Behavior: Reading and Compliance with Risk Information

We also assessed differences in safety behaviors (i.e., reading and compliance with warning information) when using CHPs (Table 3). In terms of average differences in reading warning information before use, the responsive group was the highest at 3.12, followed by the proactive, avoidance, and indifferent groups at 2.91, 2.62, and 2.51, respectively; the differences among the groups were statistically significant. Regarding differences in average values for compliance with the warning information during CHP use, the responsive group was the highest at 3.40, followed by proactive, avoidance, and indifferent groups at 3.36, 3.05, and 3.01, respectively; the differences among the groups were statistically significant. The responsive group showed a higher response rate for reading and compliance with warning information before and while using CHPs than the avoidance group, and the performance rate of the proactive group was higher than that for the indifferent group. There was no difference in reading product risk information between the indifferent and avoidance groups.

### 3.6. Factors Influencing Classification of Respondent Groups

The factors that influence the classification of RPA groups were examined (Table 4). Compared to the responsive group, those with a particularly low education level, low monthly household income level, and lower frequency of using CHPs, as well as those who did not have a child in the household, were likely to be classified in the indifferent group. Compared to the responsive group, the factors influencing the probability of being classified in the avoidance group were lower education, lower monthly household income, and being male. Compared to the responsive group, differences among factors affecting the likelihood of being classified in the proactive group were not statistically significant.

## 4. Discussion

This study sought to (a) determine whether the RPA framework could help to classify individuals into groups based on risk perceptions and efficacy beliefs about safety behaviors and (b) provide basic information to inform policies targeting each group during CHP use. We also examined factors influencing these classifications to understand the characteristics of each group. The classifications showed that the responsive group (28.1%) had the highest number of respondents, followed by the indifferent group (27.5%), avoidance group (22.4%), and proactive group (21.5%). A chi-square test of the groups for group classification was 20.8, which is statistically significant. There was a significant association between membership in one or more of the four RPA framework groups and two safety behaviors—reading and compliance with risk information—associated with CHP use. The frequency of CHP use, gender, and presence of young children in the household were statistically significant determinants of safety behavior.

The avoidance (high risk/weak efficacy) and indifferent (low risk/weak efficacy) groups had significantly lower rates of both safety behaviors than did the responsive (high risk/high efficacy) and proactive (low risk/high efficacy) groups. The variable that appeared to drive this difference in safety behaviors was a weak level of efficacy belief. In other words, a higher efficacy belief was associated with the likelihood of becoming informed and complying with risk information messages. This finding aligns with the RPA framework hypothesis, and the results of this study confirm that safety behaviors while using CHPs differ according to the type of individual. The RPA framework, which was developed to explain an individual’s attitude toward disease prevention, can be applied to safety behavior related to CHPs.

The RPA framework follows the theory of the Extended Parallel Process Model (EPPM), which considers efficacy beliefs as a moderating variable between perceived risk and health behavior [38,68]. Witte [38,68] argues that the degree of perceived risk and efficacy beliefs determine different health behaviors toward certain diseases. In the RPA model in this study, the responsive and avoidance groups showed high levels of perceived risk but engaged in different behaviors due to varying degrees of efficacy beliefs. Conversely, the proactive and indifferent groups had low perceived risk, indicating that both groups appeared to be less motivated to avoid health risks. Even if a difference in efficacy beliefs exists between the two low perceived risk groups, the RPA model assumes that there will be no difference in health behavior outcomes between the two groups. However, in this study, individuals in the proactive group showed higher frequencies in both behaviors when compared to the indifferent and avoidance groups. This result suggests that efficacy belief not only has a moderating effect on risk perception but is also the primary influence on the two safety behaviors. Some prior studies also found that efficacy belief has a primary effect [53,55,69,70]. Therefore, when developing policy, attention must be devoted to including aspects that encourage and persuade these groups to perform safety behaviors, and risk communication efforts are necessary to convey messages that enhance efficacy beliefs. Providing risk information and forming appropriate levels of risk perception is not sufficient for changing the public’s behavior. To prevent health risks caused by CHPs, individuals must believe that their own actions to understand information concerning risk are essential and, in turn, will make informed decisions based on that information.

Factors that determine types of individuals according to RPA were identified. Being in the avoidance group (high risk/weak efficacy) and indifferent group (low risk/weak efficacy) was associated with lower education and income levels. Previous studies have shown that the burden of injury is not shared equally among all societal groups, and disproportionately affects those with lower education and income levels [71,72]. Some evidence suggests that socioeconomic status associates with increased risk for accidental injury and the severity of the injury in the home [73,74]. However, relatively little attention has been paid to explaining the causal relationship between socioeconomic status and injury control/prevention. The explanatory factors that SES affects the risk of injury are complex, and psychosocial factors represent but one of many variables that can explain socioeconomic disparities in injuries [72]. Higher socioeconomic status individuals can afford resources and access to opportunity structures to develop and exercise personal efficacy [75,76]. In this study, although the causal relationship between socioeconomic status and the risk of injury was not identified, the findings provide valuable information for further investigation.

This study has several practical implications for policy and education. First, this study explored the use of the RPA framework for studying safety behaviors during CHP use. The RPA framework has been applied mainly to phenomena directly related to disease prevention, with the exception of a few studies [59,70]. The applicability of RPA to reading and compliance with risk information was confirmed in this study and suggested that this framework could be a valuable public segmentation tool to prevent injury associated with CHP use. Second, results from this study offer guidance for developing communication guidelines to establish customized CHP safety policies for groups of individuals. Based on the characteristics of each RPA group, policy effectiveness can be improved. Lastly, we identified vulnerable populations; that is, being of lower socioeconomic level, using CHPs less frequently, and the absence of young children affected both the attitudes and behaviors associated with CHP use. Korea’s CHP safety management plan is mainly developed in accordance with the product category. The results of this study suggest that shifting from product-oriented or substance-oriented CHP safety management to a ‘user-centered’ management approach would be beneficial. Therefore, prioritizing policies and communication efforts will be necessary for these vulnerable populations.

This study has some limitations. First, although several psychological and cognitive factors such as familiarity [77] or perceived risk characteristics [78] of CHPs are already known, these were not included in the analysis. Our study aimed to identify sociodemographic and socioeconomic factors, though further research is needed. Second, we could not measure the full range of CHP use frequency due to the limited length of the survey questionnaire. Finally, self-efficacy measures were targeted to general concepts of self-efficacy, rather than self-efficacy in terms of outcomes for safety behaviors. Self-efficacy, in terms of outcomes, should be considered in future research.

## 5. Conclusions

This study classified types of individuals by applying the RPA framework, considering risk perception and efficacy beliefs for the safe use of CHPs. This study identified four types of groups: Responsive, avoidance, proactive, and indifferent individuals, and applied the RPA framework to reading and compliance with risk information. Two groups characterized by weak efficacy beliefs showed lower rates of safety behavior. Therefore, the importance of strengthening efficacy belief was confirmed and provided a future direction for policies and communication. Factors influencing the division of individual types included education level, income level, frequency of CHP use, gender, and presence of young children in the household; the study also identified vulnerable populations. This research provides implications for designing risk communication messages, policies, and segmentation of individuals by risk perception and efficacy beliefs. Moreover, this research has implications for the design of risk communication messages and policies in which the importance of strengthening efficacy belief was confirmed. The results of this study suggest that a change from product-oriented or substance-oriented CHP safety management to a ‘user-centered’ management approach is needed, in addition to prioritizing policies and communication efforts that target these vulnerable populations.

## Figures and Tables

**Table 1:** Study participant characteristics.

Characteristics	Frequency	%
Gender		
Male	787	51.2
Female	750	48.8
Age		
20–29	319	21.0
30–39	320	20.8
40–49	378	24.5
50–59	374	24.3
60–65	146	9.5
Education		
Middle school graduate	51	3.3
High school graduate	634	41.2
College graduate	836	54.4
Graduate school	16	1.0
Monthly household income ^a^		
Under 299	255	16.6
300–399	374	24.3
400–499	372	24.2
500–599	323	21.0
Over 600	213	13.9
Presence of children in the household		
Yes	181	11.8
None	1356	88.2
Frequency of Chemical Household product (CHP) use	*n*	%
Rarely	181	11.8
2–3 times a month	337	21.9
1–2 times a week	533	34.7
3–4 times a week	369	24.0
5–6 times a week	74	4.8
Every day	38	2.5
More than two times a day	5	0.3

Notes: ^a^ South Korean 10,000 won (USD 1 = KRW1189.00).

**Table 2:** Respondent types based on perceived risk and efficacy beliefs.

Variables		Efficacy Beliefs		
		Low	High	χ^2^
Perceived risk	Low	Indifferent group *n* = 425 (27.7%)	Proactive group *n* = 334 (21.7%)	20.8 ***
	High	Avoidance group *n* = 344 (22.4%)	Responsive group *n* = 434 (28.2%)	

Note: ***p < 0.001.

**Table 3:** Intention to read and comply with warning information.

Respondent Groups	Reading Risk Information		Complying with Risk Information	
	Mean	S.D.	Mean	S.D.
Indifferent group	2.51 ^a^	0.81	3.01 ^a^	0.71
Avoidance group	2.62 ^a^	0.85	3.05 ^a^	0.70
Proactive group	2.91 ^b^	0.84	3.36 ^b^	0.66
Responsive group	3.12 ^c^	0.86	3.4 ^b^	0.60
F	44.32 ***		34.85 ***	

Notes: Significantly difference in post-hoc Tukey test at alpha = 0.05: a < b < c. *** *p* < 0.001.

**Table 4:** Results for multinomial logistic regression.

Variables	Indifferent (Low RP, Weak EB)		Avoidance (High RP, Weak EB)		Proactive (Low RP, High EB)	
	B	Exp(B)	B	Exp(B)	B	Exp(B)
Age	−0.01	0.99	−0.007	0.99	−0.006	0.99
Education	−0.42 **	0.66	−0.37 *	0.69	0.002	1.00
Income	−0.15 ***	0.86	−0.15 **	0.86	−0.062	0.94
CHP use frequency	−0.16 *	0.85	−0.11	0.89	−0.023	0.98
Gender (Male)	0.08	1.08	−0.39 *	0.68	0.083	1.09
Presence of child (None)	0.54 *	1.72	0.33	1.40	−0.025	0.98

Notes: Reference group = Responsive group, RP = Risk Perception, EB = Efficacy belief, B = Logistic coefficient, Exp(B) = Odds ratio, * *p* < 0.05, ** *p <* 0.01, *** *p <* 0.0001.
